# Efficacy of tracheal tube introducers and stylets for endotracheal intubation in the prehospital setting: a systematic review and meta-analysis

**DOI:** 10.1007/s00068-021-01762-5

**Published:** 2021-07-31

**Authors:** Jaden Tollman, Zubair Ahmed

**Affiliations:** 1 Institute of Inflammation and Ageing, College of Medical and Dental Science, University of Birmingham, Edgbaston, Birmingham, B15 2TT UK; 2 Surgical Reconstruction and Microbiology Research Centre, National Institute for Health Research, Queen Elizabeth Hospital, Birmingham, B15 2TH UK

**Keywords:** Endotracheal intubation, Tracheal tube introducer, Bougie, Stylet, Prehospital

## Abstract

**Purpose:**

Tracheal tube introducers and stylets remain some of the most widely used devices for aiding practitioners in performing endotracheal intubation (ETI). The purpose of this systematic review is to evaluate the efficacy of tracheal tube introducers and stylets for ETI in the prehospital setting.

**Methods:**

A literature search was conducted on the 2nd of March 2021 across PubMed, Embase (Ovid) and the Cochrane Central Register of Controlled Trials (CENTRAL) to identify relevant studies. Included studies had their data extracted and both a quality assessment and statistical analysis were performed.

**Results:**

The summary estimate of prehospital studies with video technology showed a statistically significant increase in first pass ETI success in favour of bougies (RR 1.15, CI 1.10–1.21, *p* < 0.0001). The summary estimates of prehospital studies without video technology and simulation studies with and without video technology showed no statistical difference between methods for first pass or overall ETI success. Some of the highest success rates were recorded by devices that incorporated video technology. Stylets lead to a shorter time to ETI while bougies were easier to use. Neither device was associated with a higher rate of ETI complications than the other.

**Conclusion:**

Both tracheal tube introducers and stylets function as efficacious aids to intubation in the prehospital environment. Where video technology is available, bougies could offer a statistically significant advantage in terms of first pass ETI success. Where video technology is unavailable, a combination of clinical scenario, practitioner expertise and personal preference might ultimately guide the choice of device.

**Supplementary Information:**

The online version contains supplementary material available at 10.1007/s00068-021-01762-5.

## Introduction

Endotracheal intubation (ETI) can be a life-saving advanced airway management technique for critically ill or injured patients. It remains a technically difficult but vital skill for prehospital care providers, especially for physician-led teams that regularly encounter airway compromise [1, 2]. It is not without risk, as significant morbidity and mortality can result when ETI is performed poorly [3]. Success is unsurprisingly more common amongst experienced personnel [3], highlighting a need for devices that might improve success and reduce adverse events among less experienced personnel, or particularly difficult airways.

Tracheal tube introducers (commonly referred to as bougies) and stylets are two of the most widely used devices that aid practitioners in ETI. Bougies can be passed under full or partial vision or even blindly through the vocal cords, providing a scaffold that the endotracheal tube (ETT) can be easily passed over before the bougie is withdrawn [4]. They vary in size, shape, structure and thickness and may be single-use or reusable, angulated or straight, soft tip, hard tip or even hollow-core [4]. Stylets can be inserted into the ETT before intubation, allowing it to be moulded into a shape that facilitates easier ETI before being removed as soon as the cords are passed [4]. These are also manufactured in various forms similar to bougies [4].

Clinical practice varies between healthcare systems and there is no universal consensus on when and how these two devices should be implemented into airway management protocols. In the emergency department (ED), success rates for ETI can be as high as 99% with first pass rates of 80–90% [5]. Although ETI is used in EDs to secure patient airways, a Cochrane review in 2018 found no difference between ETI and other airway securing strategies for reducing death or injury and indicated better studies were needed to make conclusive statements [6]. Both the Difficult Airway Society and American Society of Anesthesiologists currently recommend that intubating bougies and stylets may form part of a difficult airway intubation strategy [7, 8], while the Resuscitation Council UK believe a bougie should always be available for prehospital ETI [9]. A previous systematic review and meta-analysis was conducted that sought to compare the efficacy of both devices in patients undergoing ETI, though only one prehospital study was included [10]. These results may, therefore, not be generalisable to the vastly different prehospital environment.

The aim of this study was to systematically review the literature relevant to the use of bougies and stylets for ETI in the prehospital setting. This should inform a conclusion regarding their prehospital efficacy and allow practitioners to decide whether and how best to incorporate these devices into their practice. The primary outcomes of this systematic review are first pass ETI and overall ETI success. Secondary outcomes include time to ETI, ease-of-use and ETI complications such as oesophageal intubation.

## Methods

### Literature search

We used the search strategies recommended for the Preferred Reporting Items for Systematic Reviews and Meta-Analysis (PRISMA) statement [11]. We conducted a search on the 2nd of March 2021 across the following databases: PubMed, Embase (Ovid) and the Cochrane Central Register of Controlled Trials (CENTRAL). The search terms used were as follows: “Prehospital” AND (“Tracheal tube introducer” OR “Gum elastic bougie” OR “Flexible tip bougie” OR “Eschmann tracheal tube introducer” OR “Frova introducer” OR “Stylet”). No publication date limitations were set.

### Inclusion and exclusion criteria

Studies were included if they met the following criteria: (1) clinical trials and observational studies that examine the use of laryngoscopes with tracheal tube introducers and/or stylets by prehospital personnel in a prehospital setting/simulation; (2) publications from any date and country; (3) publications in English; (4) human participants of all ages and simulation models of any type.

Studies were excluded on the following basis: (1) clinical trials and observational studies that examine the use of laryngoscopes with tracheal tube introducers and/or stylets by hospital personnel in a hospital setting/simulation; (2) reviews, guidelines, editorials, letters, case reports, conference abstracts and animal studies (3) publications not in English language.

### Data collection process

Both authors independently performed the literature search and titles and abstracts were manually screened followed by removal of duplicates. Screened articles then underwent an independent full-text assessment for eligibility by both reviewers. The reference lists of included articles were screened again for additional studies that may have been missed. Any discrepancies were resolved by discussion between the authors.

### Data extraction

We extracted the following data: (1) characteristics of included studies; (2) first pass ETI success and overall ETI success of included studies.

### Quality analysis

The National Institutes of Health (NIH) quality assessment tool for observational cohort and cross-sectional studies was used independently by both reviewers (J.T., and Z.A.) to assess the methodological quality of included studies [12]. This tool assessed 14 criteria including study objectives, population, sample, exposures, outcomes and analyses. Any discrepancies were resolved by discussion between the authors.

### Statistical analysis

Data were analysed using Review Manager 5.4 (The Cochrane Collaboration, Oxford, UK). Dichotomous outcomes were analysed using risk ratios (RR) as summary statistics. The effect sizes were reported as weighted mean differences and the precision of effects sizes were reported at a confidence interval (CI) of 95%. A pooled estimate of RRs and weighted mean differences were computed using the DerSimonian and Laird random effects model [13]. Statistical significance was indicated by *p* < 0.05 or 95% CI. Statistical heterogeneity and inconsistency in treatment effects across studies were evaluated using Cochrane *Q* test and *I*^*2*^ statistics, respectively. Statistical significance was set at *p* < 0.10 for the Cochrane *Q* test. Statistical heterogeneity across studies was assessed using the *I*^*2*^ test (total outcome variability across studies).

## Results

### Study selection

**Fig. 1:**
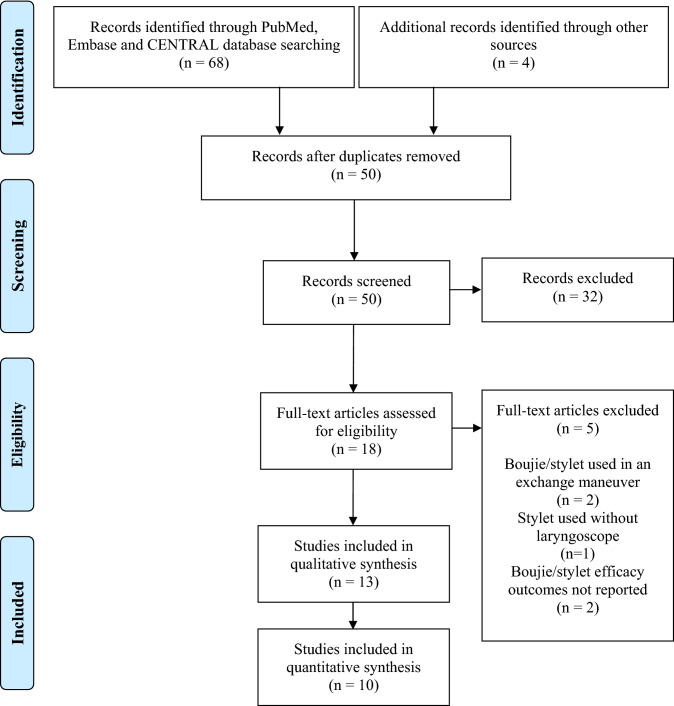
PRISMA flowchart summarising study selection process

Our search strategy identified a total of 68 articles across the PubMed, Embase (Ovid) and CENTRAL databases. Following removal of 22 duplicates, 46 articles were screened by title and abstract, of which 32 were excluded. The remaining 14 articles had their full-texts assessed for eligibility. Five full-text articles were excluded, two reported the use of a bougie/stylet in an exchange manoeuvre, one reported the use of a stylet without laryngoscope and two did not report bougie/stylet efficacy outcomes at all. The nine remaining studies had their reference lists screened and a further four studies were identified and included. A total of 13 studies were therefore included in our qualitative synthesis [14–26]. Of these studies, 10 were included in our quantitative synthesis [14, 15, 19–26]. This study selection process has been summarised in a PRISMA flow chart (Fig. 1).

### Study characteristics

Five of the studies took place in the prehospital setting [14–16, 20, 21], while eight had a design that attempted to simulate the prehospital setting [17–19, 22–26]. Six studies took place in the United States of America (USA) [14, 18–20, 23, 26] followed by two in France [16, 21], two in Australia [22, 25], one in Finland [15], one in Turkey [17] and one in Poland [24]. Each study examined various intervention combinations of direct or video ETI assisted by different types of bougies and stylets. Studies reported a variety of relevant outcomes including first pass ETI success, overall ETI success, time to ETI, ease-of-use and ETI complications. A full overview of the characteristics of included studies is given in Table 1.

**Table 1:** Study characteristics and outcomes of included studies

Study	Design	Country	Participants	Relevant Interventions	Relevant Outcomes
Bonnette et al. [14]	Secondary analysis of cluster-crossover trial, prehospital	USA	1227 adult patients	Bougie-assisted direct/video ETI, non-bougie-assisted direct/video ETI	First pass ETI success, overall ETI success, number of ETI attempts, total time to ETI/abandonment of efforts, 72-h survival, return of spontaneous circulation, hospital survival, hospital survival with favourable neurological status
Ångerman et al. [15]	Retrospective observational before-and-after study, prehospital	Finland	787 adult patients	New protocol of bougie-assisted video ETI, historical protocol of bougie-/stylet-assisted direct/video ETI	First pass ETI success, overall ETI success
Jabre et al. [16]	Retrospective observational study, prehospital	France	1442 adult patients	Bougie-assisted direct ETI, non-bougie-assisted direct ETI	First pass ETI success, overall ETI success, ETI difficulty
Karaca et al. [17]	Randomised crossover trial, simulation	Turkey	38 paramedics	Moving/stationary: stylet-assisted direct ETI, bougie-assisted direct ETI	Overall ETI success, number of ETI attempts, time to pass through vocal cords, time until cuff inflation, time until first ventilation, ease-of-use
Phelan et al. [18]	Prospective non-blinded paired study, simulation	USA	96 prehospital care providers	Stylet-assisted direct ETI, bougie-assisted direct ETI	Overall ETI success
Cooney et al. [19]	Randomised crossover trial, simulation	USA	81 ALS EMTs	Stylet-assisted direct ETI, video-stylet-assisted direct ETI	First pass ETI success, overall ETI success, number of ETI attempts, first pass ETI time, total time to ETI, satisfaction, perceived usefulness
Heegaard et al. [20]	Prospective randomised trial, prehospital	USA	51 patients ≥ 12 years old	Bougie-assisted direct ETI, non-bougie-assisted direct ETI	First pass ETI success, number of ETI attempts, total time to ETI, complications
Combes et al. [21]	Prospective observational study, prehospital	France	2674 adult patients	Bougie-assisted direct ETI, non-bougie-assisted direct ETI	First pass ETI success, overall ETI success, number of ETI attempts, complications
Woollard et al. [22]	Randomised crossover trial, simulation	Australia	79 student/qualified prehospital care providers	Stylet-assisted direct ETI, stylet-assisted Airtraq ETI	First pass ETI success, overall ETI success, complications, ease-of-use
Messa et al. [23]	Randomised crossover trial, simulation	USA	35 prehospital care providers	Stylet-assisted direct ETI, bougie-assisted direct ETI	Overall ETI success, total time to ETI, ease-of-use
Evrin et al. [24]	Randomised crossover trial, simulation	Poland	46 paramedics	(Standard) bougie-assisted direct ETI, (new) bougie-assisted direct ETI, non-bougie-assisted direct ETI	First pass ETI success, overall ETI success, total time to ETI, ease-of-use
Gregory et al. [25]	Randomised crossover trial, simulation	Australia	65 prehospital care providers	Stylet-assisted direct ETI, Portex (single-use) bougie-assisted direct ETI, Frova (single-use) bougie-assisted direct ETI, Portex (reusable) bougie-assisted direct ETI	Overall ETI success, total time to ETI, complications, ease-of-use
Le et al. [26]	Prospective cohort study, simulation	USA	35 paramedics	Bougie-assisted direct ETI, non-bougie-assisted direct ETI	Overall ETI success

*ETI* Endotracheal intubation, *ALS* Advanced life support, *EMT* Emergency medical technician

### Quality analysis

**Fig. 2:**
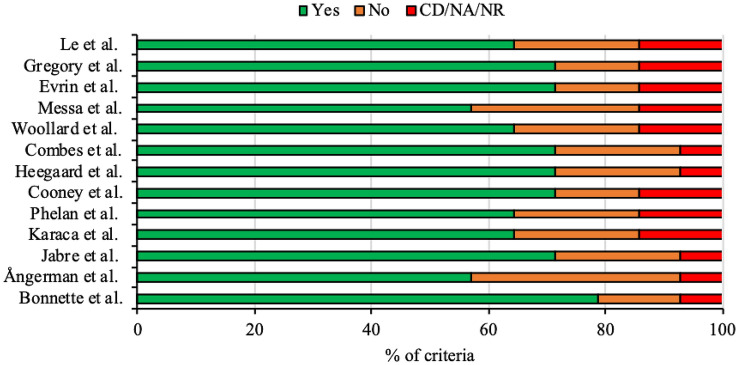
Summary of NIH quality assessment tool for observational cohort and cross-sectional studies results

Figure 2 summarises the results from our quality analysis of included studies using the NIH quality assessment tool for observational cohort and cross-sectional studies. For each study, a number of “yes”, “no”, or “cannot determine (CD)/not applicable (NA)/not reported (NR)” answers were assigned depending on whether each of the tool’s 14 criteria were satisfied. None of the studies were assigned an answer of “yes” for all 14 criteria. Bonnette et al. [14] had the highest proportion (79%) of criteria answered “yes” (i.e. high quality) while Ångerman et al. [15] and Messa et al. [23] had the lowest proportion (57%) answered “yes”. Ångerman et al. [15] also had the highest proportion (36%) of criteria answered “no” while Bonnette et al. [14], Cooney et al. [19], Evrin et al. [24] and Gregory et al. [25] all had the lowest proportion (14%) answered “no”. Every study had at least 7% of criteria answered “CD/NA/NR” as every study investigated an exposure that could not vary in amount or level. Eight studies had 14% of criteria answered “CD/NA/NR” [17–19, 22–26] as it could not be determined whether the participation rate of eligible persons was at least 50%.

### Review of primary outcomes

Table 2 summarises the primary outcomes of first pass ETI success and overall ETI success for studies with a prehospital design without video technology [16, 20, 21]. Jabre et al. [16] was the only study that did not fully report on first pass ETI success. The highest first pass ETI success rate was reported by Combes et al. [21] at 74% when a non-bougie-assisted direct ETI method was implemented. Combes et al. [21] also reported the lowest first past ETI success rate at just 2% when a bougie-assisted direct ETI method was used. Neither Jabre et al. [16] nor Heegaard et al. [20] fully reported on overall ETI success. Combes et al. [21] reported the highest overall ETI success rate with their non-bougie-assisted direct ETI method at 90.3%. The lowest overall ETI success rate of 75.5% was again reported by Combes et al. [21] using a bougie-assisted method of direct ETI.

**Table 2:** First pass ETI success and overall ETI success of prehospital studies without video technology

Study	First pass ETI success (%)		Overall ETI success (%)	
Jabre et al. [16]	Bougie-assisted direct ETI	Non-bougie-assisted direct ETI	Bougie-assisted direct ETI	Non-bougie-assisted direct ETI
	58.5	Not reported	80.5	Not reported
Heegaard et al. [20]	Bougie-assisted direct ETI	Non-bougie-assisted direct ETI	Bougie-assisted direct ETI	Non-bougie-assisted direct ETI
	70.0	65.0	Not reported	Not reported
Combes et al. [21]	Bougie-assisted direct ETI	Non-bougie-assisted direct ETI	Bougie-assisted direct ETI	Non-bougie-assisted direct ETI
	2.0	74.0	75.5	90.3

*ETI* Endotracheal intubation

Table 3 summarises the primary outcomes of first pass ETI success and overall ETI success for studies with a prehospital design with video technology [14, 15]. Both studies fully reported on first pass ETI success. Ångerman et al. [15] reported the highest first pass ETI success rate of 98.2% when their new protocol of bougie-assisted video ETI was implemented. While Bonnette et al. [14] reported the lowest first pass ETI success rate of 43.8% using a non-bougie-assisted direct/video ETI method. Both studies also fully reported on overall ETI success. Ångerman et al. [15] reported the highest overall ETI success rate at 99.6% using their new protocol of bougie-assisted video ETI. Again, Bonnette et al. [14] reported the lowest overall ETI success rate of 49.1% using a method of non-bougie-assisted direct/video ETI.

**Table 3:** First pass ETI success and overall ETI success of prehospital studies with video technology

Study	First pass ETI success (%)		Overall ETI success (%)	
Bonnette et al. [14]	Bougie-assisted direct/video ETI	Non-bougie-assisted direct/video ETI	Bougie-assisted direct/video ETI	Non-bougie-assisted direct/video ETI
	52.1	43.8	56.1	49.1
Ångerman et al. [15]	New protocol of bougie-assisted video ETI	Historical protocol of bougie-/stylet-assisted direct/video ETI	New protocol of bougie-assisted video ETI	Historical protocol of bougie-/stylet-assisted direct/video ETI
	98.2	85.7	99.6	99.2

*ETI* Endotracheal intubation

Table 4 summarises the primary outcomes of first pass ETI success and overall ETI success for studies with a simulation design without video technology [17, 18, 23–26]. Only Evrin et al. [24] fully reported on first pass ETI success, with a highest rate of 91.3% for their new model bougie-assisted direct ETI method and lowest rate of 23.9% for their non-bougie-assisted direct ETI method. All six studies fully reported on overall ETI success. The highest overall ETI success rates of 100% were reported by Karaca et al. [17] for both their moving and stationary bougie-assisted direct ETI methods, and Evrin et al. [24] for both their standard and new model bougie-assisted direct ETI methods. Gregory et al. [25] reported the lowest overall ETI success rate at just 8% when using a Portex (reusable) bougie for direct ETI.

**Table 4:** First pass ETI success and overall ETI success of simulation studies without video technology

Study	First pass ETI success (%)								Overall ETI success (%)					
Karaca et al. [17]	Moving stylet-assisted direct ETI	Stationary stylet-assisted direct ETI			Moving bougie-assisted direct ETI		Stationary bougie-assisted direct ETI		Moving stylet-assisted direct ETI	Stationary stylet-assisted direct ETI		Moving bougie-assisted direct ETI		Stationary bougie-assisted direct ETI
	Not reported	Not reported			Not reported		Not reported		97.3	97.3		100.0		100.0
Phelan et al. [18]	Stylet-assisted direct ETI				Bougie-assisted direct ETI				Stylet-assisted direct ETI			Bougie-assisted direct ETI		
	Not reported				Not reported				66.7			71.9		
Messa et al. [23]	Stylet-assisted direct ETI				Bougie-assisted direct ETI				Stylet-assisted direct ETI			Bougie-assisted direct ETI		
	Not reported				Not reported				77.0			94.0		
Evrin et al. [24]	(Standard) bougie-assisted direct ETI			(New) bougie-assisted direct ETI		Non-bougie-assisted direct ETI			(Standard) bougie-assisted direct ETI		(New) bougie-assisted direct ETI		Non-bougie-assisted direct ETI	
	73.9			91.3		23.9			100.0		100.0		86.9	
Gregory et al. [25]	Stylet-assisted direct ETI		Portex (single-use) bougie-assisted direct ETI		Frova (single-use) bougie-assisted direct ETI			Portex (reusable) bougie-assisted direct ETI	Stylet-assisted direct ETI	Portex (single-use) bougie-assisted direct ETI		Frova bougie-assisted direct ETI		Portex (reusable) bougie-assisted direct ETI
	Not reported		Not reported		Not reported			Not reported	57.0	30.0		27.0		8.0
Le et al. [26]	Bougie-assisted direct ETI				Non-bougie-assisted direct ETI				Bougie-assisted direct ETI			Non-bougie-assisted direct ETI		
	Not reported				Not reported				97.0			97.0		

*ETI* Endotracheal intubation

Table 5 summarises the primary outcomes of first pass ETI success and overall ETI success for studies with a simulation design with video technology [19, 22]. Both studies fully reported on first pass ETI success. Cooney et al. [19] reported the highest first pass ETI success rate of 96.3% using a method of video-stylet-assisted direct ETI. While the lowest first pass ETI success rate of 0% was reported by Woollard et al. [22]. Both studies also fully reported on overall ETI success. Again, Cooney et al. [19] reported the highest overall ETI success rate of 100% using a method of stylet-assisted direct ETI. While Woollard et al. [22] reported the lowest overall ETI success rate of 30% using their method of stylet-assisted direct ETI.

**Table 5:** First pass ETI success and overall ETI success of simulation studies with video technology

Study	First pass ETI success (%)		Overall ETI success (%)	
Cooney et al. [19]	Stylet-assisted direct ETI	Video-stylet-assisted direct ETI	Stylet-assisted direct ETI	Video-stylet-assisted direct ETI
	95.1	96.3	100.0	98.8
Woollard et al. [22]	Stylet-assisted direct ETI	Stylet-assisted Airtraq ETI	Stylet-assisted direct ETI	Stylet-assisted Airtraq ETI
	0.0	44.0	30.0	78.0

*ETI* Endotracheal intubation

**Fig. 3:**
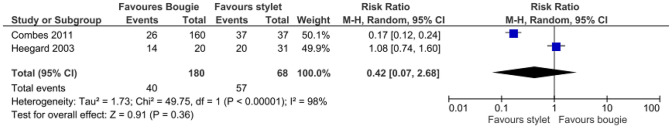
Forest plot of comparison between bougie versus stylet; outcome, first pass success rate in studies without video technology

Two studies [20, 21] were included in our meta-analysis of bougie versus stylet first pass ETI success for prehospital studies without video technology (Fig. 3) The primary outcome of first pass ETI success was chosen for this analysis as overall ETI success was not fully reported in two of the prehospital studies without video technology [16, 20]. Statistical heterogeneity across studies was high (*I*^*2*^ = 98%). The results of Combes et al. [21] favoured the use of a stylet (RR 0.17, CI 0.12–0.24), while Heegard et al. [20] did not show a statistically significant difference between devices (RR 1.08, CI 0.74–1.60). Overall, the summary estimate of prehospital studies without video technology showed no statistically significant difference between devices for first pass ETI success (RR 0.42, CI 0.07–2.68, *p* = 0.36).

**Fig. 4:**
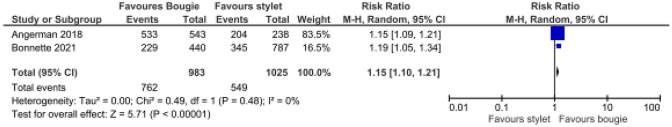
Forest plot of comparison between bougie versus stylet; outcome, first pass success rate in studies with video technology

Two studies [14, 15] were included in our meta-analysis of bougie versus stylet first pass ETI success for prehospital studies with video technology (Fig. 4). Statistical heterogeneity across studies was low (*I*^*2*^ = 0%). The results of Bonnette et al. [14] favoured the use of a bougie (RR 1.19, CI 1.05–1.34), while Ångerman et al. [15] did not show a statistically significant difference between devices (RR 1.15, CI 1.09–1.21). Overall, the summary estimate of prehospital studies with video technology showed a statistically significant difference between devices for first pass ETI success, favouring the use of a bougie (RR 1.15, CI 1.10–1.21, *p* < 0.00001).

**Fig. 5:**
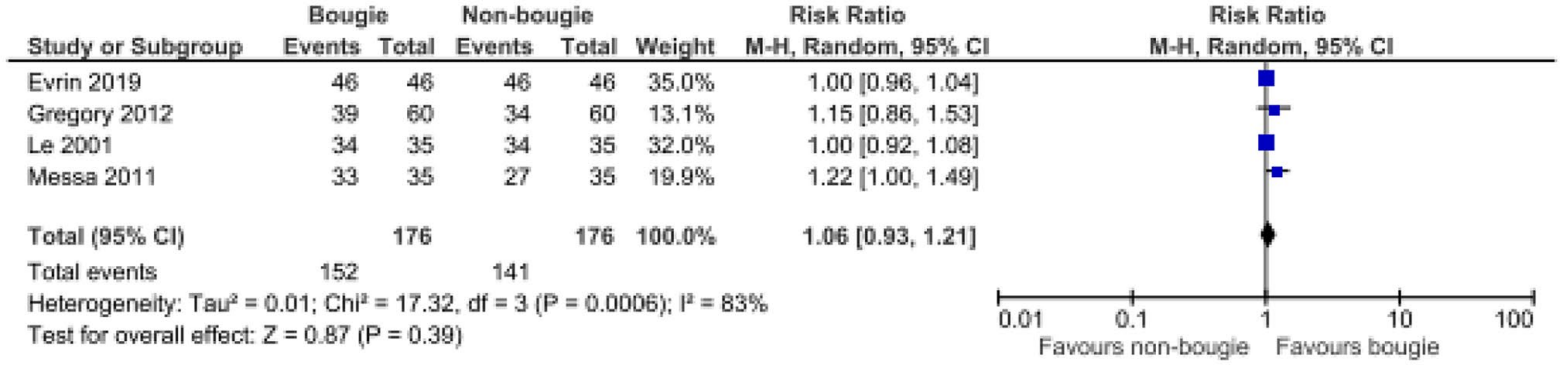
Forest plot of comparison between bougie versus stylet; outcome, overall success rate in studies without video technology

Four studies [23–26] were included in our meta-analysis of a bougie versus non-bougie method overall ETI success for simulation studies without video technology (Fig. 5). The primary outcome of overall ETI success was chosen for this analysis as first pass ETI success was not fully reported in five of the simulation studies without video technology [17, 18, 23, 25, 26]. Statistical heterogeneity across studies was high (*I*^*2*^ = 83%). All four studies [23–26] showed no statistical difference between ETI methods. Overall, the summary estimate of simulation studies without video technology showed no statistical difference between methods for overall ETI success (RR 1.06, CI 0.93–1.21, *p* = 0.39).

**Fig. 6:**
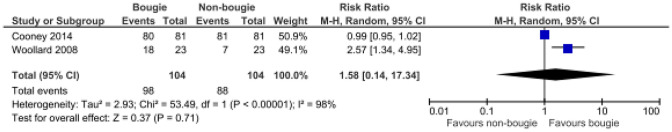
Forest plot of comparison between bougie versus stylet; outcome, overall success rate in studies with video technology

Two studies [19, 22] were included in our meta-analysis of bougie versus non-bougie method overall ETI success for simulation studies with video technology (Fig. 6). Statistical heterogeneity across studies was again high (*I*^*2*^ = 98%). The results of Woollard et al. [22] favoured the use of a bougie (RR 2.57, CI 1.34–4.95), while Cooney et al. [19] showed no statistical difference between methods (RR 0.99, CI 0.95–1.02). Overall, the summary estimate of simulation studies with video technology showed no statistical difference between methods for overall ETI success (RR 1.58, CI 0.14–17.34, *p* = 0.71).

### Review of secondary outcomes

Seven studies reported on time to ETI [14, 17, 19, 20, 23–25]. Bonnette et al. [14] reported statistically significant results that using a bougie with either direct or videolaryngoscopy led to a longer time to ETI compared to a stylet [13.0 min vs 11.0 min, HR 0.63, 95% CI 0.45–0.90). Karaca et al. [17] supports this, noting a significantly shorter duration with a stylet to pass the vocal cords in a stationary ambulance, to inflate the ETT cuff in both stationary and moving ambulances and to first ventilate in a moving ambulance. Gregory et al. [25] also concurs, reporting a significantly shorter time to ETI with a stylet compared to all three assessed bougies, none of which had a statistically significant difference in time to ETI between them. Conversely, Evrin et al. [24] was the only study that reported statistically significant results that bougies led to a shorter median intubation time, with the shortest time offered by the new model of bougie at 29 s (IQR: 25–38). Both Heegard et al. [20] and Messa et al. [23] reported no statistically significant differences in average or median time to ETI between stylets and bougies. Cooney et al. [19] noted no significant difference in median time to ETI between classic and video stylets.

Five studies reported on ease-of-use [17, 22–25]. Messa et al. [23] used a Likert ease-of-use survey and reported that overall, a significant majority of participants perceived a bougie to be easier to use than a stylet. Similarly, Evrin et al. [24] used an ease-of-use visual analogue scale (VAS) and reported that participants found bougies to be easier to use, with new model bougies being easier than standard model bougies. However, Gregory et al. [25] also used an ease-of-use VAS and noted that participants found the Portex reusable bougie to be the most difficult to use of all devices. Though the other two bougies and stylet being assessed did not vary in difficulty [25]. Karaca et al. [17] was the only study to report no significant differences in perceived ETI difficulty using a bougie versus a stylet in both a moving and stationary ambulance. Comparing stylets alone, Woollard et al. [22] used an ease-of-use VAS and reported both students and practitioners found using a stylet with an Airtraq laryngoscope for ETI to be significantly easier than using a stylet with a standard Macintosh laryngoscope.

Four studies reported on ETI complications [20–22, 25] with oesophageal intubation being the most frequently reported. Combes et al. [21] recorded a 52% rate of early intubation-related complications when a bougie was used for ETI, though they did not report a rate for when a bougie was not used. Oesophageal intubation accounted for the largest proportion of these complications at 36%, followed by arterial oxygen desaturation at 26% [21]. Gregory et al. [25] concluded that use of the Portex reusable bougie in particular was associated with the highest risk of oesophageal placement. The other two bougies and stylet that were being assessed all had a similar risk to one another [25]. However, Heegard et al. [20] did not report a significant difference in oesophageal intubation rate between a bougie (5%) and non-bougie (6.5%) method of ETI. When just a stylet is used, Woollard et al. [22] reported a lower rate of oesophageal intubations when used alongside an Airtraq laryngoscope as opposed to a standard Macintosh laryngoscope. This was true for both students and experienced practitioners, whereby practitioners recorded zero oesophageal intubations with the Airtraq and stylet combination.

## Discussion

Our systematic review qualitatively and quantitatively integrates the literature relevant to the use of bougies and stylets for ETI in the prehospital setting. It is clear that both bougies and stylets should have a role in prehospital ETI, though bougies appear to offer a statistically significant increase in first pass ETI success when used alongside video technology. Additionally, there are suggestions that particular devices may convey advantages in certain clinical situations. These devices, as well as prehospital environment considerations, practitioner experience and study design quality all necessitate consideration when choosing between bougies or stylets.

Our results suggest that bougies offer a statistically significant increase in first pass ETI success when used alongside video technology in the prehospital setting. Though, conclusions inferred from this isolated statistically significant result must be considered in the context of high study heterogeneity and the multiplicity of comparisons being made. Nevertheless, some of the highest first pass and overall ETI success rates in both prehospital and simulation studies incorporated some form of video technology. Ångerman et al. [15] achieved this in the prehospital setting by utilising a Frova intubating introducer alongside a C-MAC videolaryngoscope. Alternatively, Cooney et al. [19] achieved their high first pass success in the simulation setting by utilising the Clarus Video System (CVS), a video-assisted semi-rigid fibreoptic stylet, alongside direct laryngoscopy. Improvements in ETI success with videolaryngoscopy are not a novel concept and have been previously demonstrated by another systematic review in the hospital environment [27]. The advantage that video-assistance conveys might be more notable and prove particularly useful with novice intubators [28]. Newer technologies such as the CVS then widen the scope of how we might install video technology into pre-existing devices such as the simple malleable stylet. When working with these devices, however, consideration must be given towards the cost that is inevitably higher than in their technologically sparse counterparts. Still, whether incorporated into the analogous laryngoscope or the stylet/bougie itself, our review highlights that video technology might offer the key to further improving upon ETI success in the prehospital setting. In particular, where video technology is available, our findings suggest that some practitioners may wish to opt for a bougie over a stylet to maximise their first pass ETI success.

Our review of secondary outcomes suggests that stylets offer speed while bougies offer ease-of-use, though neither device appears to lead to a higher rate of complications than the other. Again, conclusions inferred from these results must be considered in the context of high study heterogeneity and the multiplicity of comparisons being made. Still, where video technology is unavailable, bougies may have a role where the intubating practitioner is less experienced, while stylets may have a role in cases where catastrophic airway compromise is imminent. However, it is worth noting the results of Gregory et al. [25] reported that the Portex reusable bougie was both the most difficult to use and led to the most complications while having the lowest overall ETI success across simulation studies. A practitioner’s specific preference of bougie might therefore instead be towards the Portex single-use or Frova intubating introducer. Overall, these aspects must be considered on a case by case basis in the context of a markedly diverse and fast changing prehospital environment. Confronted with a patient with significant inhalation injury who is rapidly losing an airway, the practitioner may opt for the superior speed of a stylet to secure it as soon as possible. However, in a scenario where the practitioner is significantly less experienced, the ease-of-use of a bougie might facilitate success. Thus, where video technology is unavailable, the choice of device might be dictated by a combination of the clinical scenario, practitioner expertise and personal preference. This is in accordance with the results of Sheu et al.’s systematic review and meta-analysis [10].

The issue of practitioner experience has been touched upon and demands further discussion as it is clearly able to significantly influence success with devices assessed in this study. Models of prehospital care and their respective expertise vary across the world, with mainland Europe favouring physician-led services while the UK is predominantly paramedic- and technician-led [29]. Even compared to seemingly similar services such as the USA, there are large differences in personnel competencies, with more comprehensively trained US paramedics recruited at a higher level than in the UK [29]. Therefore, prehospital care systems in different countries may report varying degrees of success with the same array of ETI devices, simply by virtue of their prehospital personnel training and experience with ETI. Participant intubators across our studies varied from physicians, paramedics and emergency medical technicians to even include students. Some of our studies acknowledged and attempted to account for this by recording participants’ previous ETI experience and familiarity with the devices being assessed. In line with this, it is integral that pre-existing participant ETI experience is treated as a potential confounding variable in future studies, particularly when the make-up of prehospital care teams has proven to be so varied.

A mixture of both simulation and real-world prehospital studies were incorporated into our study. We felt that this was necessary to capture a wider range of data due to a lack of real-world prehospital studies, reflecting the distinct challenges involved with research in this setting. A possible solution to this lies with high-quality simulation, perhaps including dedicated advanced prehospital simulation laboratories [30]. By developing high-quality simulation, we can overcome the obstacles of research in the prehospital context (such as lack of control), while still being able to study realistic prehospital scenarios complete with serious game-inspired techniques and methods [30]. This would facilitate the generation of much needed prehospital-oriented randomised controlled trials (RCTs) that can be systematically reviewed to inform guidelines. Without simulation, the power of reviews such as ours becomes handicapped by the methodological quality of the few prehospital studies we can analyse.

It should finally be noted that our study has considered the efficacy of bougies/stylets in the context of their conventional and direct use in facilitating ETI. Two studies were excluded during the selection process that utilised bougies in an exchange manoeuvre, converting from one type of airway to another [31, 32]. One study even used a lighted stylet device for ETI without the aid of a laryngoscope [33]. The efficacy of either of these methods cannot be validated nor revoked by our study. However, readers should have an awareness that the utility of bougies/stylets may not be limited to a singular ETI technique as in this review.

## Limitations

This study is limited in the specific device comparisons that can be made due to the sheer number and variety of bougies/stylets currently in clinical use or trials. Within the realms of video-assisted ETI alone, variations in software can significantly change a device’s performance. Drawing absolute conclusions concerning efficacy has, therefore, proven problematic and general trends and themes have instead been explored. Having such a wide variety of devices available may appear beneficial to prehospital services, though it poses difficulties for synthesis of comprehensive clinical guidelines. This may ultimately lead to ambiguity in clinical decisions regarding what device to use and when. Pre-existing experience of participant intubators also limit the conclusions that can be drawn regarding the efficacy of any one device. Aforementioned variations in prehospital provision across the world mean that efficacy of a device tested in the USA may not be comparable to its efficacy in France and vice versa [29]. Future studies must account for this and an internationally standardised method of assessing a prehospital practitioner’s ETI capabilities should be employed. Lastly, this review is limited by a distinct lack of RCTs, with a heavy reliance on observational studies. Sheu et al.’s systematic review and meta-analysis conversely included RCTs only, yet three of their five trials were in a preoperative setting and one was in an ED [10]. This review aimed to focus exclusively on the prehospital efficacy of these devices to guide the practice of prehospital personnel in their unique setting, thus inclusion of hospital-based RCTs was deemed inappropriate. Despite including studies of higher evidence, Sheu et al. concurs in part with the conclusion of our review for when video technology is unavailable, that clinician expertise and personal preference should determine choice of intubating device, owing to a lack of significant differences in primary efficacy outcomes [10]. Nevertheless, the methodological robustness of some of the studies that were included in this review is questionable. Many of the studies ran with admittedly small samples while one study satisfied just 57% of our quality assessment criteria [23]. This stresses the need for greater quality control amongst prehospital studies, reinstating the importance of high-quality simulation to overcome the inherent lack of controls in a real-world prehospital setting [30].

## Conclusions

It can be concluded that both tracheal tube introducers and stylets function as efficacious aids to intubation in the prehospital environment. However, where video technology is available, bougies could offer a statistically significant advantage in terms of first pass ETI success. The overwhelming variety of devices and competency disparities across different countries’ prehospital services make a true assessment of individual efficacy challenging. While acknowledging the potentially higher costs, video-assisted devices may offer distinct advantages and techniques that incorporate this technology could be the answer to further improving ETI success. Where video technology is unavailable, a combination of clinical scenario, practitioner expertise and personal preference might be used to ultimately guide the choice of device. Future studies must account for pre-existing practitioner experience when assessing efficacy of any ETI technique and emphasis should be placed on high-quality simulation with robust quality control.

## Supplementary Information

Below is the link to the electronic supplementary material.

Supplementary file1 (DOCX 15 KB)

## Data Availability

Not applicable.
