# Supplementary material for: Efficacy of tracheal tube introducers and stylets for endotracheal intubation in the prehospital setting: a systematic review and meta-analysis
Source: Eur J Trauma Emerg Surg. 2021 Jul 31;48(3):1723–35. doi: 10.1007/s00068-021-01762-5 (PMC9192420; doi:10.1007/s00068-021-01762-5)
Supplement: Supplementary file 1 — Supplementary file1 (DOCX 15 KB) [file 68_2021_1762_MOESM1_ESM.docx]

**Supplementary Table:** Studies excluded following screening of titles and abstracts.

| Study | Reason for Exclusion |
| --- | --- |
| Akhgar et al. | Hospital personnel in a hospital setting/simulation |
| Kumar et al. | Hospital personnel in a hospital setting/simulation |
| Timmermann | Review |
| Soleimanpour et al. | Irrelevant |
| Daniel et al. | Case report |
| Szarpak et al. | Irrelevant |
| Picard et al. | Case report |
| Leeuwenburg | Irrelevant |
| Sime et al. | Case report |
| Ridgway et al. | Irrelevant |
| Butchart et al. | Irrelevant |
| Sudrial et al. | Case report |
| Rusan et al. | Irrelevant |
| Combes et al. | Case report |
| Morris et al. | Case report |
| Nocera | Case report |
| Wray et al. | Irrelevant |
| Voroba et al. | Hospital personnel in a hospital setting/simulation |
| Everhart et al. | Review |
| Dabrowski et al. | Irrelevant |
| Choi et al. | Irrelevant |
| Jaubert et al. | Conference abstract |
| Bernhard et al. | Irrelevant |
| Bernhard et al. | Irrelevant |
| Hossfeld et al. | Irrelevant |
| Escott et al. | Irrelevant |
| Vranckx et al. | Review |
| Xue et al. | Letter |
| Butchart et al. | Irrelevant |
| Lewis et al. | Irrelevant |
| Jaber et al. | Hospital personnel in a hospital setting/simulation |
| Hwang et al. | Irrelevant |
